# Iodine and Bromine Analysis in Human Urine and Serum by ICP-MS, Tailored for High-Throughput Routine Analysis in Population-Based Studies

**DOI:** 10.3390/analytica7010006

**Published:** 2026-01-06

**Authors:** Thieli Schaefer Nunes, Lucas Schmidt, Kayla Peterson, Rosalind Wright, Julio Alberto Landero-Figueroa

**Affiliations:** Department of Environmental Medicine, Icahn School of Medicine at Mount Sinai, New York, NY 10029, USA

**Keywords:** halogens, bromine, bromide, iodine, iodide, ICP-QQQ, ICP-MS, urine, serum

## Abstract

Iodine is essential for thyroid hormone synthesis and is particularly critical during pregnancy, where excess and mainly its deficiencies can impair fetal neurodevelopment and increase maternal complications. Bromine has also gained attention due to its potential to interfere with iodine metabolism and contribute to adverse health effects when present in excess. Monitoring iodine and bromine in biological samples, especially urine and serum, is therefore important for assessing thyroid function and population health. This work presents a simple and robust ICP-MS method for simultaneous determination of bromine and iodine in urine and serum. The procedure uses a 20-fold dilution with 10 mmol L^−1^ ammonia containing 0.1% (*w*/*w*) EDTA-2Na, ensuring solution stability, minimizing sample-to-sample variability, and eliminating the need for matrix-matched calibration. EDTA-2Na effectively prevents precipitation of metal species at high pH, avoiding blockages in the sample introduction system. Method accuracy was confirmed through certified reference materials and spike-recovery experiments, both showing suitable agreement for the two analytes. Precision was consistently strong (RSD < 6%), and low detection limits were achieved (0.78 μg L^−1^ for Br and 0.24 μg L^−1^ for I). The use of a high-efficiency nebulizer enabled analysis with only 50 μL of sample, making the method suitable for limited-volume specimens. Overall, this approach provides a sensitive, accurate, and practical solution for large-scale population studies and clinical applications.

## 1. Introduction

Iodine plays a crucial role in maintaining human health, with its significance under-scored by its essential function in thyroid hormone synthesis [1]. The thyroid gland relies on iodine to produce hormones crucial for regulating metabolism, growth, and development. Particularly noteworthy is its critical role during pregnancy, where adequate iodine intake becomes paramount for ensuring optimal fetal brain development and overall maternal health. Deficiencies in iodine during pregnancy can lead to a range of adverse outcomes, including impaired cognitive function in offspring and increased risk of complications during childbirth. Although iodine deficiency remains a major public health concern, excessive iodine intake is considerably less common but may still disrupt thyroid function, particularly in susceptible populations [1,2]. Consequently, monitoring iodine levels within the body emerges as a crucial aspect of healthcare, especially among vulnerable populations such as pregnant women.

Furthermore, studies regarding bromine levels in the human body have gained attention in recent years, firstly, due to recent research indicating that bromine also plays an essential role in the biological system, acting in the formation of collagen IV [3-5]. Secondly, due to its antagonistic behavior with iodine, an excess of bromine in the body can significantly affect iodine metabolism by decreasing iodide accumulation in the thyroid, which leads to hypothyroidism, and, consequently, further contributes to the growing interest in this area of research in the last few years [6,7]. The measurement of bromine levels holds significant importance due to its potential impact on iodine metabolism. Therefore, iodine status and metabolism are not solely influenced by iodine intake but are also affected by the intake and retention of goitrogenic halides, notably bromide [6]. Excessive intake of bromide, arising from certain foods, environmental sources, and brominated compounds used in industrial or pharmaceutical applications, can result in tissue accumulation and toxic effects known as “bromism” [8]. Common manifestations of bromism include neurological disturbances such as headache, fatigue, confusion, and dizziness, which may progress to more severe symptoms including hallucinations, delirium, and seizures. Additionally, bromism can affect the gastrointestinal system, leading to nausea, vomiting, and abdominal pain. Chronic exposure to bromine also may result in the development of bromoderma, a dermatological condition characterized by a rash resembling acne, as well as mucous membrane irritation [8]. Furthermore, the integrity of critical organs such as the thyroid and mammary glands can be compromised by excess of Br, underscoring the necessity of monitoring bromine levels alongside iodine for comprehensive assessment of thyroid health and overall well-being [6,9]. In a recent study conducted by Novakova et al. [7] in 2023, the behavior of iodine and bromine in hemodialysis patients was investigated in comparison to patients with normal renal function (control group). The study revealed that iodine levels did not exhibit significant changes between the two groups. However, a notable finding was the discovery that serum bromine levels were 75% lower than those observed in the control group. Despite this substantial decrease, the authors emphasized that there is limited research on the potential essentiality of bromine and this requires further investigation into its role and implications, but that this Br decreasing behavior may be associated with fatigue and sleep disturbances that affect hemodialysis patients.

Therefore, exploring iodine and bromine levels as a biomarker through population-based studies can offer invaluable insights into broader public health concerns. Based on that, the determination of bromine (Br) and iodine (I) levels in biological samples, particularly urine and serum, presents significant challenges and opportunities for scientific research in this area, mainly for population-based study [7,10].

Urine iodine concentration (UIC) is the most commonly used indicator to assess iodine nutritional status in populations. UIC is favored due to the ease of sample collection and the ability to access a larger volume of samples compared to serum samples, which require a more invasive collection method and yield smaller volumes. Nevertheless, UIC can present some challenges regarding some variabilities such as water intake, diet, and pregnancy, which may significantly affect the results. So, to mitigate these effects the UIC are usually adjusted for urinary creatinine concentration. However, factors such as ethnicity, age, muscle mass, and protein intake can influence urinary creatinine levels [11]. Despite these challenges, some works have demonstrated that measuring UIC across 24 h urine collections can enhance the accuracy of assessing iodine nutritional status [12-14]. On the other hand, many studies report that serum iodine concentration (SIC) can be a better biomarker of iodine metabolism, since there is no free iodine in blood; iodine is rapidly captured by thyroid cells, and because of that SIC can directly reflect the bioavailable I in the thyroid [2,11,14,15]. Therefore, both matrices provide complementary insights into dietary intake, uptake, metabolism, and temporal dynamics [2,16]. Moreover, integrating information from both types of analyses can provide a more comprehensive understanding of Br and I status and its metabolism.

One of the most common methods for analyzing iodine in urine is based on the Sandell–Kolthoff reaction (S–K) method [17]. This method involves an initial digestion step to eliminate interfering substances, followed by spectrophotometric or colorimetric detection using the S–K reaction. Furthermore, the S–K method is labor-intensive and time-consuming, involving significant chemical risks, including the use of hazardous substances like arsenic and cerium and it is applicable only for iodine in urine [18]. Consequently, it is not suitable for bromide analysis, nor is serum an appropriate matrix for this method, un-like urine. Despite the development of various methods that focus on iodine and bromine determination in biological samples over recent decades, ICP-MS has emerged as the most common alternative to them. Therefore, the ICP-MS technique offers great advantages; it stands out for its high sensitivity, selectivity, and precision, making it an invaluable tool for quantifying trace levels of bromine and iodine in biological samples [19,20]. Additionally, ICP-MS allows the possibility of isotopic analysis of trace levels of iodine-129, which is a long-lived radioisotope that is produced naturally by the nuclear interaction of cosmic rays with xenon in the upper atmosphere and also by the nuclear fission of U and Pu [21-27], further enhancing its versatility and utility in research and analytical applications. However, sample preparation is generally required, and it is a crucial step in ICP-MS analysis, especially for halogen determination. One promising approach involves sample extraction using alkaline solutions [10,19,20], which can keep the halogens stable in solution, avoiding possible losses of halogen species (e.g., I_2_, HI, Br_2_ and HBr) due the low pH [16,19,28]. Most of the recent works usually use tetramethylammonium hydroxide (TMAH) or ammonia (NH_3_) solution as the alkaline medium for the samples, calibration solutions and rinse solution in order to keep the halogens stable and minimizing memory effects in ICP-MS system [29-31]. However, to the best of our knowledge, most existing studies focus on specific analytical aspects of I and Br determination in urine and serum, with limited emphasis on simple and robust methods designed for large-scale population-based applications.

Another crucial aspect to consider in developing a method for subsequent application in medical studies is determining the minimum appropriate sample volume required, mainly because many of these studies rely on archived bio specimens on which different types of analysis are intended for the same sample. Methods that underestimate or over-estimate sample consumption can, respectively, limit or render certain studies unfeasible and, for that reason, an adequate sample sizing is essential to develop a robust method that ensures the possibility of carrying out reanalysis and/or new types of analyses [21,32-36].

Therefore, the goal of this work was to develop and critically evaluate a simple, sensitive and robust analytical method based on alkaline dilution for bromine and iodine determination by ICP-MS applicable for urine and serum in low-sample consumption, evaluating possible analytical issues and making it applicable to be used in a population-based studies as well as a routine analysis method for public health monitoring.

## 2. Materials and Methods

### 2.1. Instrumentation

Iodine and bromine were quantified by Agilent 8900 triple quadrupole ICP-MS system (Agilent Technologies; Santa Clara, CA, USA) equipped with a Meinhard High Efficiency Concentric Nebulizer (CytoNeb 200, 200 μL min^−1^) (Golden, CO, USA), a Peltier cooled double-pass spray chamber, and a quartz torch with a quartz injector tube (2 mm i.d.). Additionally, Pt cones, the X-lenses stack, argon humidifier (70-803-1268, Elegra^™^; Melbourne, Australia), and a SPS 4 autosampler (Agilent Technologies, Santa Clara, CA, USA) were utilized. Moreover, He was used as a collision gas in the ICP-MS to remove or minimize any possible interferences such as ^40^Ar^39^K and ^40^Ar^38^Ar^1^H on ^79^Br or ^113^In^14^N, ^115^In^12^C, ^126^Te^1^H and ^87^Sr^40^Ar and ^111^Cd^16^O on ^127^I [29-31,37-39]. Operational parameters used in this study are summarized in Table 1.

### 2.2. Chemicals and Materials

The following reagents and solutions were used to set up the proposed method: ultra-pure water (Milli-Q, resistivity < 18.2 MΩ cm), 21% *w*/*w* ammonia solution (A512-P500, TraceMetal^™^ grade, Fisher Chemical; Fairlawn, NJ, USA) and disodium ethylenediaminete-traacetate dihydrate (EDTA-2Na) (E4884, Sigma-Aldrich; Burlington, MA, USA) were used to prepare the analytical solutions used in this work.

Bromine stock solution in water as solvent (1000 mg L^−1^, IC-BR-M-100, HPS; North Charleston, SC, USA) and iodine stock solution in water as solvent (1000 mg L^−1^, 41271, TraceCert^®^; Darmstadt, Germany) were used to prepare the reference standards. Furthermore, an internal standard containing Y, Rh, Ir, Sb and Te, was employed across all analytical solutions. This mixture was prepared using a commercial internal standard solution which contained Y, Rh and Ir (10 mg L^−1^, IV-61129, Inorganic Ventures; Christiansburg, VA, USA); one contained Te (1000 mg L^−1^, CGTEN1, Inorganic Ventures; Christiansburg, VA, USA) and another one contained Sb (100 mg L^−1^, MSSB-100PPM, Inorganic Ventures; Christiansburg, VA, USA).

The standard calibration solutions (containing 8 points) were made in the ranges of 0.05 to 100 μg L^−1^ (I) and 0.5 to 1000 μg L^−1^ (Br), diluted in 10 mmol L^−1^ of ammonia, 0.1% *w*/*w* EDTA-2Na and 10, 10, 10, 50 and 50 μg L^−1^ of Y, Rh, Ir, Sb and Te as ISTD, respectively. Four different urine and four different serum proficiency testing materials (PT) routinely purchased for quality control and quality assurance from the Quebec Multielement External Quality Assessment Scheme (QMEQAS) were used as samples of urine and serum in this work. Ultrapure water and a 240 mmol L^−1^ ammonia solution were used as rinse solutions between all the samples to avoid carryover.

Moreover, recovery tests were performed using spiked urine and serum samples for both analytes, as well as reference materials, including Seronorm^®^ trace elements Urine L-2 (SR210713, Seronorm^®^; Billingstad, Norway), Seronorm^®^ trace elements Serum L-2 (SR203113, Seronorm^®^; Billingstad, Norway) and SRM 2670a Toxic Elements in Urine, Low-Level (NIST; Gaithersburg, MD, USA), to evaluate the accuracy and precision of the method. For these experiments, iodine and bromine were spiked into the reference materials at concentrations of 100 and 1000 μg L^−1^, respectively. Additionally, analyte stability in analytical solution of urine and serum was assessed after 36 h of storage at room temperature (≈20 °C) and compared with results obtained from freshly prepared samples (<8 h).

### 2.3. Sample Preparation by Alkaline Dilution

A 100 μL liquid aliquot of sample (urine or serum) was transferred directly to a 5 mL polypropylene (PP) vial. Then, 1500 μL ultrapure water plus 400 μL of a diluent containing 0.5% *w*/*w* EDTA-2Na, 50 mmol L^−1^ ammonia, 50 μg L^−1^ of Y, Rh and Ir as ISTD and 250 μg L^−1^ of Te and Sb as ISTD was added to the same vial, reaching a final volume of 2 mL. The 10 mmol L^−1^ ammonia final concentration was based on previous work [10,21,31,36]. Subsequently, PP screw caps were employed to seal the PP vials containing the samples, which were then vortexed to ensure thorough mixing. The urine samples were deemed ready for analysis at this stage. However, for serum samples, an additional filtration step was added using a 5 μm nylon membrane (EW-12915-97, Cole-Parmer^®^; Vernon Hills, IL, USA) to prevent nebulizer clogging caused by possible protein precipitation from the serum. Blank samples were prepared using identical procedures described above for all experiments. The limits of detection (LOD) and quantification (LOQ) were calculated as three times and ten times the standard deviation, respectively, from 10 individual method blanks.

Furthermore, the selection of ISTD candidates was based by previous research [7,18,36,40,41] involving halogens determination by ICP-MS. Additionally, the concentrations of Te and Sb as ISTDs were adjusted to be slightly higher than those which are considered more rare to be found in biological samples (Y, Rh and Ir). This adjustment aimed to provide better contrast in scenarios where the real sample could contain significant levels of Sb and/or Te, ensuring a better robustness and suitability for these samples.

It is also important to mention that these 5 mL PP vials are directly compatible with the 60-position racks used in most ICP-MS autosamplers, so no further sample transference is needed. This compatibility reduces the need for additional sample preparation steps and minimizes the risk of cross-contamination from transfers and handling.

## 3. Results and Discussion

### 3.1. Previous Development and Optimization of the Method

At the beginning of the experiments, during method development, three main issues related to sample introduction were observed. The first issue was the formation of salt deposits after running dozens (around 60) of urine samples. These deposits were clearly visible on the nebulizer tip, quartz transfer tube, torch, and cones. To ensure a more robust method that could operate for longer periods without requiring cleaning after every batch, an argon humidifier was connected to the nebulizer gas. This modification allowed the method to run over 120 urine samples without significant salt deposit formation. Moreover, no sensitivity loss for the analytes and ISTDs was observed regarding the use of the Ar humidifier, so, its use resulted in a more robust method.

The second issue was related to precipitated proteins from the serum samples that were clogging randomly in the probe and nebulizer during the analysis. This problem was solved by filtering the samples through a 5 μm nylon filter before analysis. This step is crucial, especially because the method uses a High Efficiency Concentric Nebulizer (sample uptake ≈ 200 μL min^−1^), which has a narrower capillary than the usual nebulizer (sample uptake ≈ 400 μL min^−1^) used in ICP-MS.

The third issue was related with the insolubility of some metals due the alkaline dilution; this was confirmed as the method produced consistent results only when the samples were prepared on the same day, within a short timeframe of less than 4 h between sample preparation and analysis by ICP-MS. However, when the time interval between sample preparation and ICP-MS determination exceeded 6 h, the results became less precise and in cases where the sample was prepared the previous day, with intervals of 24 h or more, the results showed much greater variability. This variability was also reflected in the recovery profiles of the internal standards, mainly for Ir, which can form insoluble species like hydroxides in alkaline pH. Although the analytical solution contained only 10 mM NH_3_, it likely led to the formation of insoluble hydroxides of certain metals, such as Y and Ir, directly affecting their use as internal standards and potentially impacting in the sample introduction system. This is because urine can contain high concentrations of elements like Ca and Mg, which may already be present as slightly soluble or even insoluble species like hydroxides or carbonates, potentially causing partial or complete clogging in the sample introduction system, mainly in the nebulizer.

It is important to note that the precipitation of these elements as carbonates and/or hydroxides is not a rapid process and it is not easily predictable. Based in our experience, one-batch freshly prepared samples can work without observing any problems, but if the same samples are analyzed after several hours, they may start precipitate formation; the amount of precipitate will also depend on the sample itself, mainly considering urine samples. Proteins are typically more resistant to dilute alkaline solutions, but not all elements are soluble at an alkaline pH [42]. Therefore, to address these challenges, EDTA-2Na was added to the diluent solution to chelate these elements, making them soluble in the analytical solution. After the inclusion of 0.1% *w*/*w* EDTA-2Na in the final solution, the method proved to be much more robust in terms of the stability of the analytical solutions and the precision of the results throughout the analytical sequence. The concentration of EDTA-2Na added was based on a recent study published by Rosen et al. [43], which also observed similar challenges related to the clogging of the ICP-MS sample introduction system when alkaline solutions were used for further iodine determination.

After implementing these optimizations (using an argon humidifier in the nebulization system, a filtration step for serum samples, and adding EDTA to the diluent solution) no further issues with sample introduction system blockages were observed. Additionally, there were no significant differences in recovery rates among the internal standards in both matrices. This suggests that the variations in ISTD recovery rates observed during method development were mainly due to solubility issues due the absence of EDTA in the diluent solution.

**Figure 1.**
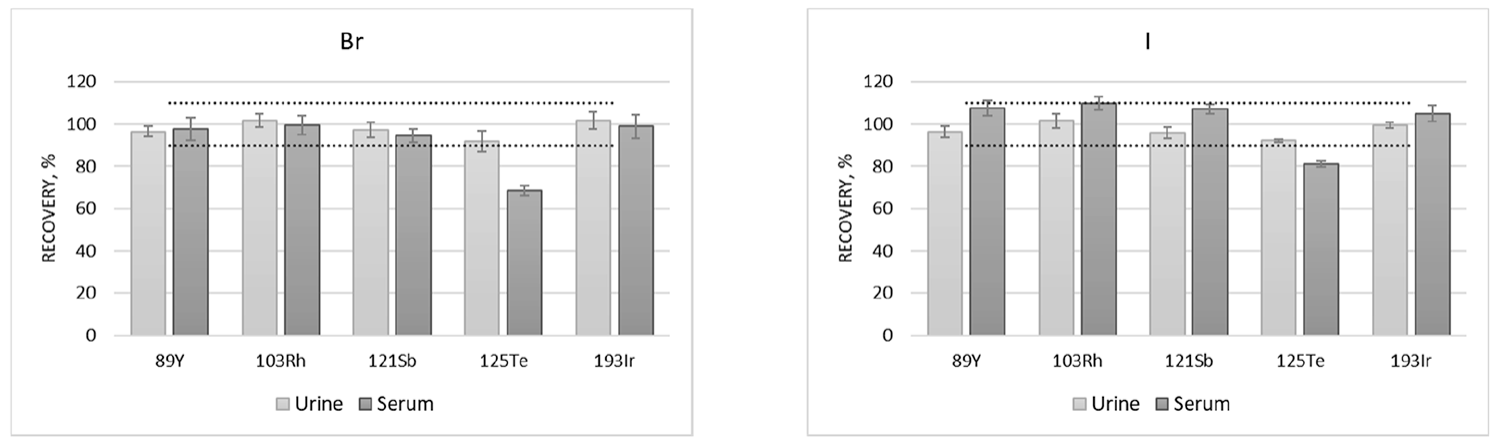
Recoveries of Br and I from the spiked samples of urine and serum using Y, Rh, Sb, Te and Ir as ISTD. The dashed lines are included as reference lines to improve contrast and facilitate relative comparison among recovery values. Spiked urine sample = QM-U-Q2405 and spiked serum sample = QM-S-Q2307 (see Supplementary Material for more details).

Furthermore, as shown in Figure 1, Te was the only ISTD evaluated that did not show suitable recoveries in the serum matrix for both analytes. This discrepancy is likely due to the higher organic content in serum compared to external calibration curve and the urine samples. It is known that Te has a signal enhancement, sometimes referred to as the carbon effect, in the presence of carbon in the analytical solution; consequently, if it is used as ISTD, it can result in an underestimated concentration for analytes that do not exhibit the same carbon effect [40,44]. The external calibration curve with 0.1% (*w*/*w*) EDTA-2Na has a total carbon content of approximately 357 mg L^−1^ in the final analytical solution and a typical urine sample with about 2% urea and 0.1% creatinine prepared in this method has an expected total carbon of approximately 578 mg L^−1^ (from urine + from EDTA). In contrast, a typical serum sample with about 7% protein (mostly as albumin) results in an expected total carbon content of approximately 2214 mg L^−1^ in the final analytical solution. In this way, the carbon content of urine samples is less than twice higher than the calibration solution, whereas the carbon content of serum samples is over six times higher. Therefore, except for Te, all other ISTDs evaluated in this work were suitable for the determination of I and Br in these samples.

However, Ir was chosen as the ISTD due to its greater rarity in biological samples compared to Te and Sb. Although Y (6.2 eV) and Rh (7.5 eV) are also very rare in these matrices, Ir (9.0 eV) has an ionization potential closer to I (10.4 eV) and Br (11.8 eV). Moreover, Ir showed the best recovery results considering the two matrices and both analytes. Therefore, Ir was selected as the ISTD to ensure a greater method robustness.

### 3.2. Performance of Analytical Method

Firstly, to better evaluate the present analytical method, some figures of merit based on the ICH M10 guidelines were applied in this study. The limit of detection (LOD) and quantification (LOQ) for I and Br using this present method are shown in Table 2. These values were determined by analyzing 10 individual method blanks according to the sample preparation procedure.

Based on the LOD and LOQ, it is evident that this method, despite having a dilution factor of 20, demonstrates excellent sensitivity suitable for its intended purpose, as shown in Table 2. According to the World Health Organization (WHO), iodine levels in urine below 100 μg/L are classified as indicative of iodine deficiency. These levels are further categorized by their risk of severity for hypothyroidism: severe (<20 μg/L), moderate (20–49 μg/L), and mild (50–99 μg/L) [45]. Although this classification specifically pertains to urine samples, it can be reasonably extrapolated to serum samples, as the concentrations of iodine and bromine are expected to be of similar magnitudes in both matrices [46,47]. Furthermore, previous studies have shown that bromine levels in urine and serum are typically from one up to two orders of magnitude higher than iodine levels [7,46]. Consequently, the calibration curve range for bromine in this study was set ten times higher than that for iodine. Therefore, an LOD of 0.24 μg/L for iodine and 0.78 μg/L for bromine in urine or serum sample are adequate for monitoring the analytes in these samples at concentrations relevant to nutritional assessment and environmental or dietary exposure studies.

To investigate the precision and the accuracy of this method, some serum and urine proficiency testing materials plus some reference materials of urine and serum as well as some spiked samples were analyzed in triplicate and these results are presented in Tables 3 and 4.

Precision (RSD) according to the Horwitz equation [48,49], as well as suitable agreements to the reference values were obtained for both analytes (when applicable) in urine and serum. It is possible to observe that all results showed an RSD of less than 6% for both analytes and in both matrices. In addition, due to the absence of a certified reference value for Br in this type of samples, spike and recovery test for Br and I were performed in both matrices.

Additionally, a mass variation test (ranging from 50% to 150%) was conducted in this study to evaluate the robustness of the method against variations in the matrix, such as salt and organic matter content. According to *t*-test for comparison of two experimental means (two tailed distribution) no significant differences (*p* > 0.05) were observed between the results using 100% of the recommended mass and those using less (LM) or more matrix (MM), as shown in Tables 3 and 4. This also demonstrated that the selection of Ir as ISTD for both Br and I was a suitable choice for this method.

Table 5 summarizes the results obtained from evaluating the stability of the analytical solution by analyzing four different samples of each matrix after 36 h at room temperature. The results demonstrated good precision and agreement (*p* > 0.05) with those from fresh sample preparation (within 8 h). This indicates that the method exhibits strong robustness concerning the stability of the analytical solution from a sample preparation which does not have a matrix decomposition step.

It is also important to mention that this method was capable of processing a batch of 120 samples in less than 10 h. Throughout the analysis, blank samples, quality control samples (QCs), reference materials (RMs), and in-house materials from urine and serum were integrated into the batch sequence. These were positioned after the calibration curve, every 20 samples, and at the end of the sequence to ensure adequate quality control for each bracket size.

This method is characterized by low sample consumption and reduced dilution, resulting in a small final analytical volume and minimal waste generation. Specifically, only 0.1 mL of sample into 2 mL final volume is required, which is substantially lower than the volumes reported in other methods with a similar approach [10,18,36,41]. To accommodate this, a low-sample consumption nebulizer was used, as detailed in the instrumentation section, requiring less than 360 μL analytical solution per run and requiring only 100 μL of sample. This setup offers significant advantages, such as the ability to reanalyze samples up to four additional times if needed or even allows for the possibility of scaling down the sample preparation to a final volume of 1 mL and spending only 50 μL of sample and one extra reanalysis if necessary. This is particularly crucial when dealing with limited or valuable samples, where acquiring additional material for repeat analyses may not be possible.

In this context, Table 6 provides an overview of previously reported ICP-MS methods and the proposed approach for iodine and bromine determination in biological matrices. As summarized, these previous methods employ alkaline or acidic diluents and single-quadrupole ICP-MS instrumentation, often optimized for a single matrix or analyte and requiring relatively large sample volumes (0.2–1.0 mL). Although alkaline reagents and EDTA have been individually reported, the present method uniquely integrates a unified low-volume sample preparation (0.1 mL), a single dilution protocol applicable to both urine and serum, and ICP-QQQ operation under helium collision mode with argon humidifier. This combination enhances robustness against matrix-related and polyatomic interferences, particularly for Br, while minimizing sample handling and preparation complexity. Importantly, the proposed approach achieves limits of detection comparable to, or lower than, those reported previously, despite the reduced sample volume and simplified workflow. Together, the comparison in Table 6 demonstrates that the novelty of the present method lies in its integrated analytical strategy and practical suitability for routine clinical and large-scale population-based studies. Therefore, all the precautions implemented here generated a very simple, versatile and steady method for further Br and I determination in urine and serum samples.

## 4. Conclusions

The integration of a single analytical method for Br and I determination in both serum and urine samples by ICP-MS has proven to be highly effective for the purpose of this work. This method, employing Ir as ISTD, an alkaline dilution approach with the addition of EDTA and a 20-fold dilution factor with reduced sample utilization, demonstrated exceptional robustness concerning the stability of analytical solutions, variations in sample mass, and analysis time.

The use of a small volume of alkaline solution as a diluent offered significant advantages, including reduced waste generation, minimized exposure to ammonia vapors during sample preparation and no memory effect during the analysis process. Additionally, incorporating 0.1% *w*/*w* EDTA-2Na was crucial to prevent the precipitation of insoluble metal hydroxides during analysis, thereby enhancing the stability of the analytical process. This resulted in a simpler, more versatile, and more applicable method for population-based studies as well as a routine analysis method for public health monitoring.

In this regard, using this strategy made it possible to achieve a simple, robust, precise, accurate, and sensitive method, with LOD and LOQ well below the recommended levels for studies aiming to evaluate Br and I in urine and serum. Furthermore, the use of a high-efficiency nebulizer in the ICP-MS system facilitated sample preparation for both matrices with volumes as low as 100 μL, and even 50 μL, while still allowing for reanalysis of the same analytical solution. This is particularly advantageous when dealing with limited or valuable samples, where obtaining additional material for repeated analyses may not be feasible.

## Supplementary Material

Supplemental material

**Supplementary Materials:** The following supporting information can be downloaded at https://www.mdpi.com/article/10.3390/analytica7010006/s1, Table S1: Results of precision and recovery in the spiked urine (QM-U-Q2405) and serum (QM-S-2307) samples.

## Figures and Tables

**Table 1.** ICP-MS operating conditions.

Parameters	Value
RF Power	1550 W
Nebulizer gas	0.90 L min^−1^
Nebulizer pump	0.10 rps (using 0.76 mm i.d. tubing) ≈200 μL min^−1^ sample uptake
Makeup gas	0.37 L min^−1^
Spray chamber	2 °C
Collision/reaction cell	4 mL min^−1^ (He)
Acquisition mode	3 replicates, 30 sweeps/replicate
Q1 → Q2, dwell time	^127^I → ^127^I (Analyte), 0.70 s ^89^Y → ^89^Y (ISTD), 0.15 s ^103^Rh → ^103^Rh (ISTD), 0.15 s ^121^Sb → ^121^Sb (ISTD), 0.15 s ^125^Te → ^125^Te (ISTD), 0.15 s ^193^Ir → ^193^Ir (ISTD), 0.15 s ^79^Br → ^79^Br (Analyte), 0.15 s

**Table 2.** Limit of detection and quantification expressed in the analytical solution (μg L^−1^) and in sample (urine or serum) (μg L^−1^).

Element	In Analytical Solution ^a^		In Sample ^a,b^	
	LOD	LOQ	LOD	LOQ
Br	0.04	0.13	0.78	2.6
I	0.01	0.04	0.24	0.79

a Obtained from method blanks (*n* = 10)

b Considering 20-fold dilution factor of the sample (urine or serum).

**Table 3.** Results of bromine (Br) in urine and serum samples (*n* = 3).

Sample	Result (μg L^−1^)	Rec. (%)	Acceptance Requirements	
	Mean ± SD (RSD, %)		Reference Values	RSD (%) ^a^
URINE				
NIST 2670a	1727 ± 35 (2.1)	-	-	10
Urine L-2 (Seronorm^™^)	1096 ± 26 (2.4)	86.7	1264 ^c^	10
QM-U-Q2324	3193 ± 107 (3.3)	-	-	9
QM-U-Q2404	1201 ± 13 (1.1)	-	-	10
QM-U-Q2406	1231 ± 28 (2.3)	-	-	10
QM-U-Q2405	1780 ± 50 (2.8)	-	-	10
QM-U-Q2405 (50% LM) ^b^	1690 ± 72 (4.3)	-	-	10
QM-U-Q2405 (50% MM) ^b^	1834 ± 69 (3.8)	-	-	10
Spike Test (in QM-U-Q2405) ^d^	1016 ± 40 (4.0)	101.6	1000	11
SERUM				
Serum L-2 (Seronorm^™^)	548 ± 27 (5.0)	79.6	689 ^c^	12
QM-S-Q2307	2258 ± 30 (1.3)	-	-	9
QM-S-Q2308	2623 ± 47 (1.8)	-	-	9
QM-S-Q2407	3111 ± 43 (1.4)	-	-	9
QM-S-Q2408	2729 ± 36 (1.3)	-	-	9
QM-S-Q2308 (50% LM) ^b^	2655 ± 87 (3.3)	-	-	9
QM-S-Q2308 (50% MM) ^b^	2607 ± 50 (1.9)	-	-	9
Spike Test (in QM-S-Q2407) ^d^	988 ± 57 (5.7)	98.8	1000	11

a Acceptable RSD were obtained according to the Horwitz equation

b LM = Less Matrix and MM = More Matrix

c Approximate values from Seronorm^™^

d Detailed spike test results are shown in the Supplementary Material.

**Table 4.** Results of iodine (I) in urine and serum samples (*n* = 3).

Sample	Result (μg L^−1^)	Rec. (%)	Acceptance Requirements	
	Mean ± SD (RSD, %)		Reference Values	RSD (%) ^a^
URINE				
NIST 2670a	87.8 ± 1.7 (1.9)	99.5	88.2 (87.1–89.3)	15
Urine L-2 (Seronorm^™^)	536 ± 12 (2.3)	94.9	565 (452–679)	12
QM-U-Q2324	143 ± 4 (3.0)	100.7	142 (113–171)	14
QM-U-Q2404	252 ± 3 (1.3)	100.1	252 (202–302)	13
QM-U-Q2406	113 ± 2 (1.5)	99.2	114 (90.3–138)	15
QM-U-Q2405	106 ± 3 (3.1)	98.2	108 (85.2–131)	15
QM-U-Q2405 (50% LM) ^b^	100 ± 4 (3.9)	92.6	108 (85.2–131)	15
QM-U-Q2405 (50% MM) ^b^	107 ± 1 (1.3)	99.2	108 (85.2–131)	15
Spike Test (in QM-U-Q2405) ^d^	100 ± 1 (1.4)	99.6	100	15
SERUM				
Serum L-2 (Seronorm^™^)	53.5 ± 2 (3.8)	78.6	68 ^c^	17
QM-S-Q2307	60.1 ± 0.5 (0.9)	106.3	56.5 (38–75)	16
QM-S-Q2308	86 ± 0.6 (0.7)	103.5	83.1 (59.8–106)	15
QM-S-Q2407	117 ± 3 (2.4)	106.1	110 (81.8–138)	15
QM-S-Q2408	57.8 ± 1.7 (2.9)	104.1	55.5 (37.2–73.8)	16
QM-S-Q2308 (50% LM) ^b^	81.7 ± 3.6 (4.5)	98.3	83.1 (59.8–106)	15
QM-S-Q2308 (50% MM) ^b^	89.1 ± 3.2 (3.6)	107.2	83.1 (59.8–106)	15
Spike Test (in QM-S-Q2407) ^d^	105 ± 4 (3.6)	105.1	100	15

a Acceptable RSD were obtained according to the Horwitz equation

b LM = Less Matrix and MM = More Matrix

c Approximate values from Seronorm^™^

d Detailed spike test results are shown in the Supplementary Material.

**Table 5.** Results of analytical solution stability test of urine and serum after 36 h from sample preparation, expressed as Mean ± SD (RSD, %).

Sample	Br (μg L^−1^)		I (μg L^−1^)	
	<8 h	36–48 h	<8 h	36–48 h
URINE				
QM-U-Q2324	3193 ± 107 (3.3)	3256 ± 68 (2.1)	143 ± 4 (3)	139 ± 3 (2.2)
QM-U-Q2404	1201 ± 13 (1.1)	1212 ± 27 (2.2)	252 ± 3 (1.3)	242 ± 9 (3.6)
QM-U-Q2405	1780 ± 50 (2.8)	1822 ± 60 (3.3)	106 ± 3 (3.1)	103 ± 2 (2.2)
QM-U-Q2406	1231 ± 28 (2.3)	1236 ± 35 (2.9)	113 ± 2 (1.5)	109 ± 2 (2.0)
SERUM				
QM-S-Q2307	2258 ± 30 (1.3)	2320 ± 39 (1.7)	60.1 ± 0.5 (0.9)	59.9 ± 1 (1.6)
QM-S-Q2308	2623 ± 47 (1.8)	2747 ± 89 (3.2)	86.0 ± 0.6 (0.7)	85.6 ± 0.2 (0.2)
QM-S-Q2407	3111 ± 43 (1.4)	3220 ± 97 (3.0)	117± 3 (2.4)	113 ± 1 (0.9)
QM-S-Q2408	2729 ± 36 (1.3)	2826 ± 86 (3.0)	57.8 ± 1.7 (2.9)	58.1 ± 0.2 (0.4)

**Table 6.** Overview of previously reported ICP-MS methods and the proposed approach for iodine and bromine determination in biological matrices.

Matrix	Analyte(s)	Sample Preparation (SV/DF/Dil.) ^a^	Calibration Range (μg/L) ^b^	LOD (μg/L) ^c^	Reference
Urine, Serum	I, Br	SV: 0.1 mL; DF: 20×; Dil.: 10 mM NH_3_ + 0.1% EDTA.	I: 0.05–100 Br: 0.05–1000	I: 0.24 Br: 0.78	This work
Serum	I, Br	SV: Not reported; DF: 10×; Dil.: 1% TMAH + 0.01% Triton X-100.	I: 20–200 Br: 200–2000	NR ^d^	[7]
Saliva	I, Br	SV: 0.2 mL; DF: 20×; Dil.: 25 mM NH_3_.	I: 0.1–1 Br: 1–10	I: 10 Br: 30	[10]
Urine	I	SV: 0.5 mL; DF: 10×; Dil: 1% TMAH + 0.01% Triton X-100.	I: 1–100	NR ^d^	[18]
Urine, Serum, Plasma, Breast milk	I	SV: 0.2 mL; DF: 10×; Dil.: 7 mM NH_3_ + 1.5% iPrOH.	I: 1–100	I: 0.233	[31]
Urine	I	SV: 1.0 mL; DF: 10×; Dil.: 57 mM NH_3_ + 0.1% EDTA.	I: 3–203	I: 3.3	[36]
Urine	I	SV: 0.5 mL; DF: 20×; Dil.: 1.5% HCl + Triton X-100.	NR ^d^	I: 4	[41]
Urine, Plasma	I, Br	SV: 0.2 mL; DF: 10×; Dil.: 1% HNO_3_.	I: 10–40 Br: 400–1600	I: 1.6 Br: 52	[46]

a SV: sample volume; DF: dilution factor; Dil.: diluent

b Calibration range expressed in analytical solution

c LOD expressed in sample

d NR = Not reported.

## Data Availability

The original contributions presented in this study are included in the article/Supplementary Material. Further inquiries can be directed to the corresponding authors.

## Abbreviations

The following abbreviations are used in this manuscript:

Br: Bromine
Dil: Diluent
DF: Dilution Factor
I: Iodine
ICP-MS: Inductively Coupled Plasma Mass Spectrometry
ICP-QQQ: Triple Quadrupole ICP-MS
iPrOH: Isopropanol
LOD: Limit of detection
LOQ: Limit of quantification
NR: Not reported
Rec: Recovery
RSD: Relative Standard Deviation
SD: Standard Deviation
SV: Sample Volume
TMAH: Tetramethylammonium Hydroxide
