# Supplementary material for: Iodine and Bromine Analysis in Human Urine and Serum by ICP-MS, Tailored for High-Throughput Routine Analysis in Population-Based Studies
Source: Analytica (Basel). Author manuscript; Available in PMC 2026 Apr 23. (PMC13101949; doi:10.3390/analytica7010006)
Supplement: Supplemental material [file NIHMS2158605-supplement-Supplemental_material.pdf]

Table S1. Results of precision and recovery in the spiked urine (QM-U-Q2405) and serum (QM-S-2307) samples.

Analyte	Spike value (μg L^{-1})	Measured value (μg L^{-1})[a]			Recovery (%)	Acceptance requirements[b]	
		Unspiked sample	Spiked sample	Spike value		RSD (%)	Recovery (%)
**URINE**							
Br	1000	1780 ± 50 (2.8)	2797 ± 40 (1.4)	1016 ± 40 (4.0)	101.6	11	80 - 110
I	100	106 ± 3 (3.1)	206 ± 1 (0.7)	100 ± 1 (1.4)	99.6	15	80 - 110
**SERUM**							
Br	1000	2258 ± 30 (1.3)	3246 ± 57 (1.8)	988 ± 57 (5.7)	98.8	11	80 - 110
I	100	60.1 ± 0.5 (0.9)	165 ± 4 (2.3)	105 ± 4 (3.6)	105.1	15	80 - 110

[a]Results expressed as Mean ± SD (RSD, %) for n = 3; [b]Acceptable requirements were obtained according to the Horwitz equation.
